# Balloon dilation versus endoscopic stricturotomy in the treatment of Crohn’s anastomotic stricture: experimental randomized study

**DOI:** 10.1007/s00464-026-12700-0

**Published:** 2026-03-16

**Authors:** Ondrej Ryska, Martin Lukas, Martin Kolar, Jaroslav Kalvach, Marketa Lengalova, Aneta Tremerova, Jan Ptacnik, Stefan Juhas, Jana Juhasova

**Affiliations:** 1 PIGMOD Center, Laboratory of Cell Regeneration and Plasticity, Institute of Animal Physiology and Genetics, Czech Academy of Sciences, Libechov, Czech Republic; 2 Royal Lancaster Infirmary, University Hospitals of Morecambe Bay NHS Foundation Trust, Lancaster, UK; 3 Lancaster University Medical School, 9 Standen Park House, Lancaster, LA1 3FF UK; 4 IBD Clinical and Research Center, ISCARE a.s., Prague, Czech Republic; 5 Department of Surgery, Military University Hospital and 2nd Faculty of Medicine, Charles University, Prague, Czech Republic; 6 Department of Surgery, University Hospital Královské Vinohrady, Prague, Czech Republic; 7 First Surgical Clinic of Thoracic, Abdominal and Injury Surgery, General University Hospital and First Faculty of Medicine, Charles University, Prague, Czech Republic

**Keywords:** Crohn’s disease, Anastomotic stricture, Balloon dilation, Stricturotomy, Porcine model

## Abstract

**Background:**

Up to 80% of patients with Crohn’s disease (CD) require surgery, and anastomotic strictures are a frequent postoperative complication with limited response to medical therapy. Endoscopic balloon dilation (EBD) and endoscopic stricturotomy (ESt) are widely used minimally invasive treatments, though comparative evidence remains scarce. Standardized animal models enable controlled assessment of therapeutic efficacy. This study compared EBD and ESt in a randomized experimental model replicating CD-associated anastomotic strictures.

**Methods:**

A chemically induced anastomotic stricture model was established in miniature pigs using a standardized ileo-sigmoid side-to-side anastomosis followed by serial TNBS/phenol injections. Forty-four animals with confirmed strictures were randomized to EBD, ESt, or control groups. Interventions consisted of graded balloon dilation or needle-knife stricturotomy with clip closure. Stricture diameter was measured endoscopically at baseline and every 8 weeks for 6 months. Primary outcomes were change in anastomotic diameter and long-term luminal patency. Secondary outcomes included adverse events and animal welfare indicators.

**Results:**

Both EBD and ESt increased anastomotic diameter compared with baseline. Significant improvement occurred only after ESt (final diameter 17.51 ± 4.71 mm vs. 11.27 ± 1.46 mm at baseline; *p* = 0.0002). EBD produced a non-significant trend toward enlargement (16.09 ± 5.35 mm; *p* = 0.0554). Diameter gain over 6 months was significantly greater with ESt than EBD (6.24 ± 4.40 mm vs. 3.31 ± 5.01 mm; *p* = 0.042). Controls showed no meaningful change. Three perforations occurred (two EBD, one ESt), all treated endoscopically without mortality. Weight gain and overall health status were comparable across groups throughout follow-up.

**Conclusion:**

In this randomized experimental study, both EBD and ESt were technically feasible and safe. ESt resulted in significantly greater and more durable luminal enlargement than EBD. These findings support stricturotomy as a potentially more effective endoscopic option for fibrostenotic CD and may help reduce or delay surgical intervention in affected patients.

It is estimated that up to 80% of patients with Crohn’s disease (CD) will eventually require surgical intervention for strictures, fistulas, or abscesses [1]. Postoperative recurrence of strictures at the anastomotic site is frequently observed [2, 3]. Anastomotic strictures develop through a combination of local and technical factors, including bacterial stasis and elevated intraluminal pressures [4]. Owing to the fibrostenotic nature of these strictures, the effectiveness of medical therapy is limited.

Although surgical management provides a more definitive solution, it carries the risk of perioperative and postoperative complications and also disease recurrence [5, 6]. Consequently, various minimally invasive endoscopic interventions—such as endoscopic balloon dilation (EBD), endoscopic needle knife stricturotomy (ESt), or self-expanding stents—have been explored [7, 8].

The therapeutic outcome is determined not only by local factors at the anastomotic site but also by the overall disease activity and the patient’s response to conservative management. Moreover, human studies remain limited with respect to the extent of longitudinal monitoring. In contrast, standardized animal models of anastomotic stricture offer an optimal setting for objective evaluation and comparative analysis of different techniques [9].

The aim of this study was to compare EBD and ESt in a randomized trial using an animal model designed to mimic anastomotic strictures associated with Crohn’s disease recurrence.

## Materials and methods

### Experimental animals

A total of 56 laboratory miniature pigs from the Institute of Animal Physiology and Genetics (Libechov, Czech Republic) weighing 48.5 ± 10.5 kg were included in the study. All animals were transported to the experimental site (Institute of Animal Physiology and Genetics, Czech Academy of Sciences, Libechov) at least 5 days prior to the start of the experiment to ensure adequate acclimatization. The experimental protocol (Nos. 40/2018 and 58/2020) was approved by the institutional expert committee of the Czech Academy of Sciences for the approval of animal experimentation projects. The project was supported by Czech health research council (NU22-08-00554).

### Model of model of Crohn's disease anastomotic stricture

To mimic anastomotic recurrence in Crohn’s disease patients after ileocecal resection, and to facilitate straightforward endoscopic access within the porcine anatomy, a side-to-side ileosigmoid anastomosis was created [9].

After intramuscular premedication with tiletamine 4 mg/kg + zolazepam 4 mg/kg (Zoletil 100; Virbac, Carros, France), ketamine 5 mg/kg (Narketan 10; Vétoquinol, Lure, France), and xylazine 1 mg/kg (Rometar 2%; Spofa, Prague, Czech Republic), general anesthesia was induced and maintained by intravenous application of 1% propofol (Propofol 1%; Fresenius Kabi, Bad Homburg, Germany) and inhalation of 2.5% isoflurane (Isoflurin 1000 mg/g; Vetpharma Animal Health, Barcelona, Spain). General anesthesia was provided using an anesthetic machine (WATO EX-65; Mindray, Shenzhen, China) in combination with a Syramed mSP6000 infusion pump (Arcomed, Kloten, Switzerland) and an iMEC10 vital signs monitor (Mindray). Analgesia was achieved by intramuscular application of flunixin meglumine (flunixin injection, 2 mL/45 kg body weight; Norbrook, Newry, Northern Ireland) and fentanyl (100 mg/pig/day, Tramadoli hydrochloridum, TRAMAL 100MG/2ML INJ SOL 5X2ML, STADA Arzneimittel AG, Germany). Thermal comfort was maintained with heating pads.

**Fig. 1:**
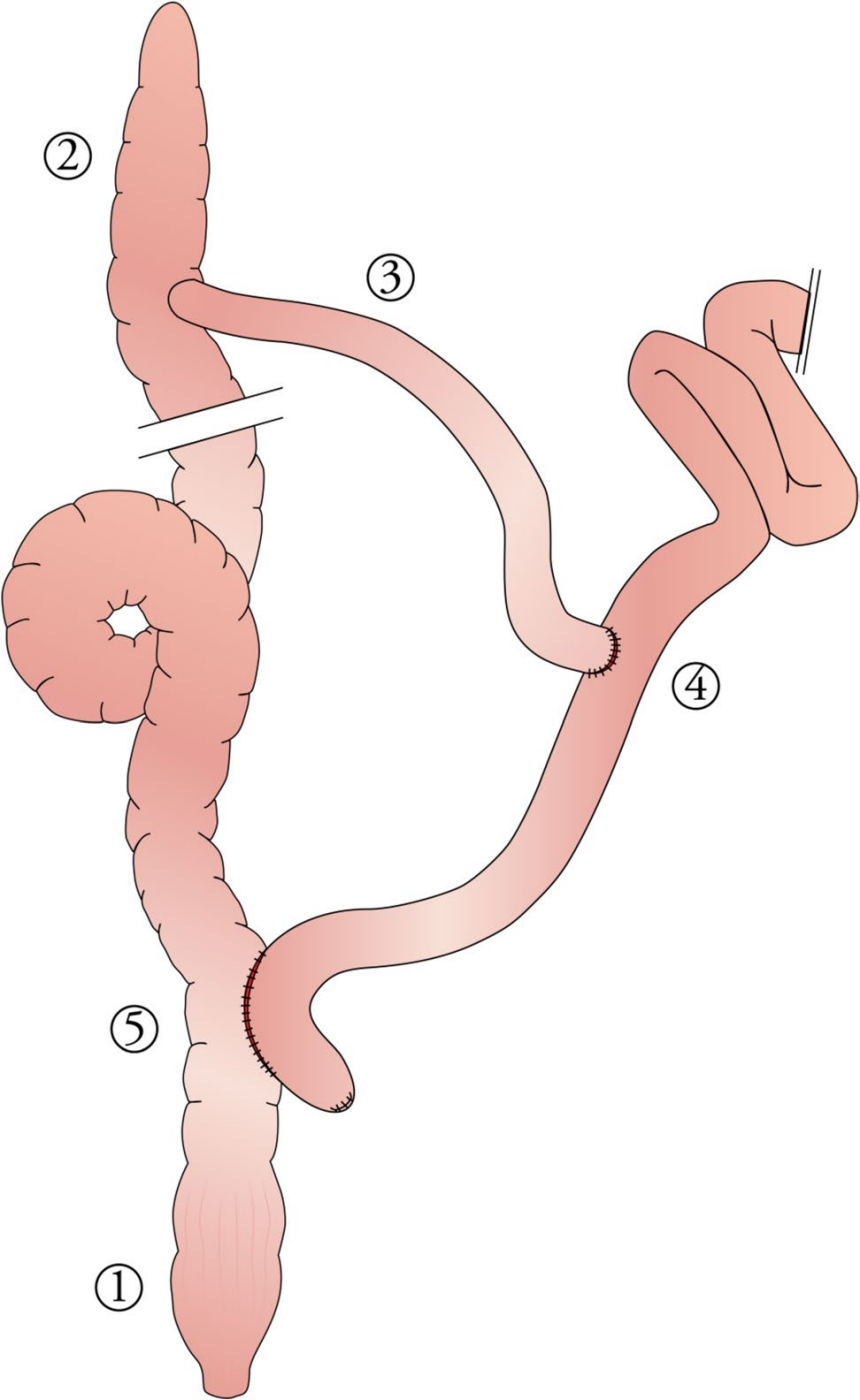
Scheme of the modified Roux-en-Y surgery performed to create accessible ileocolonic anastomosis. 1—rectum; 2—caecum; 3—terminal ileum; 4—side-to-end ileo-ileal anastomosis; 5—side-to-side ileocolonic anastomosis

**Fig. 2:**
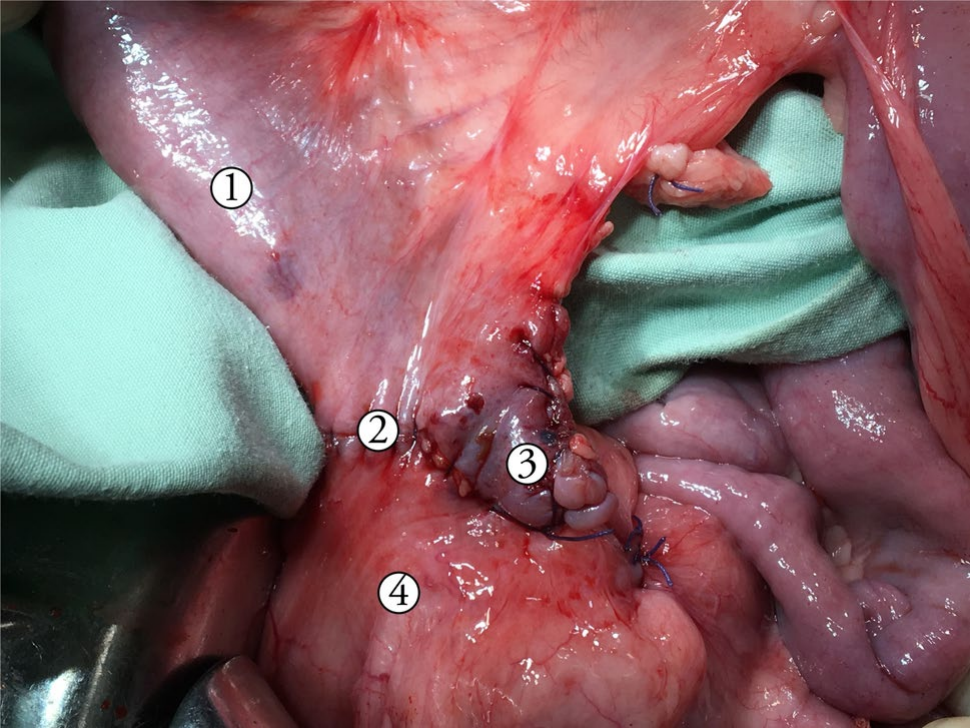
Intraoperative photo of the side-to-side ileocolonic anastomosis. 1—afferent ileal loop; 2—side-to-side anastomosis; 3—blind loop; 4—sigmoid colon

The abdominal cavity was accessed through a lower left side (Rutherford-Morrison access) laparotomy. The terminal ileum was identified and transected 20 cm proximal to the ileocecal junction. Intestinal continuity was re-established using a modified Roux-en-Y technique, consisting of a hand-sewn side-to-side ileosigmoid anastomosis positioned 20 cm from the anorectal junction, along with a side-to-end ileoileal anastomosis of the terminal ileal stump (Figs. 1 and 2). Each ileosigmoid anastomosis was standardized to an initial diameter of 20 mm, measured intraoperatively. The abdominal cavity was closed in layers: the fascia with continuous mass closure using Carprolon HRS 48 (Resorba, Nuremberg, Germany), the subcutaneous tissue with PGA Resorba HR 22 (Resorba), and the skin with Chiraflon DS 30, USP 2/0 – EP3 (CHIRANA T. Injecta, Stara Tura, Slovakia).

Two weeks after the creation of the anastomosis, endoscopic evaluation was performed under anesthesia with tiletamine, zolazepam, ketamine, and xylazine, using a GIF-XTQ160 endoscope (Olympus, Tokyo, Japan) connected to a CV-170 imaging system (Olympus). The ileosigmoid anastomosis was identified, and 2–3 mL of a solution containing 5% Liquefied phenol (> 89.0%) and 0.2% 2,4,6-trinitrobenzenesulfonic acid (TNBS) solution (both from Sigma-Aldrich, St Louis, Mo, USA) was injected into the submucosa of each quadrant using an endoscopic injection needle. This procedure was repeated every 2 weeks (at least four times) until stricture formation occurred.

### Interventions

Four weeks after the final injection, all animals were randomly allocated into study arms. Subjects assigned to the interventional groups underwent either endoscopic balloon dilation (EBD) or endoscopic stricturotomy (ESt). The remaining subjects constituted the control group and received no treatment.

Prior to each intervention, the anastomosis was evaluated and its diameter measured under the same anesthetic regimen.

**Fig. 3:**
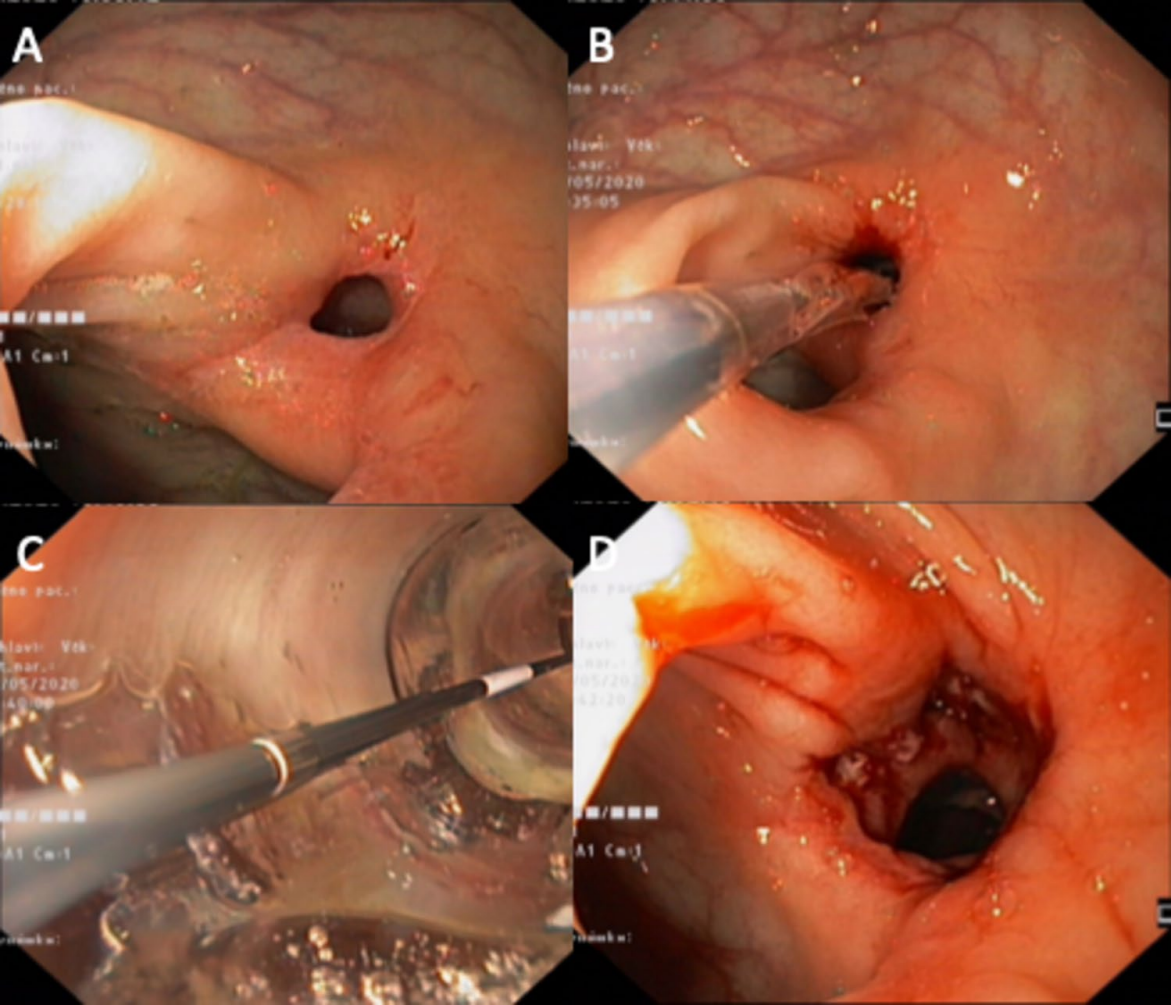
Endoscopic balloon dilation. **A** native stricture, **B** balloon insertion, **C** view thru the inflated balloon, **D** result after dilation

EBD were carried out using standard endoscopic balloons (length 55 mm, diameter 18 mm; Olympus). The anastomotic site was gradually dilated by applying pressures ranging from 1.5 to 5.5 atmospheres, with each inflation maintained for 2 min (Fig. 3).

**Fig. 4:**
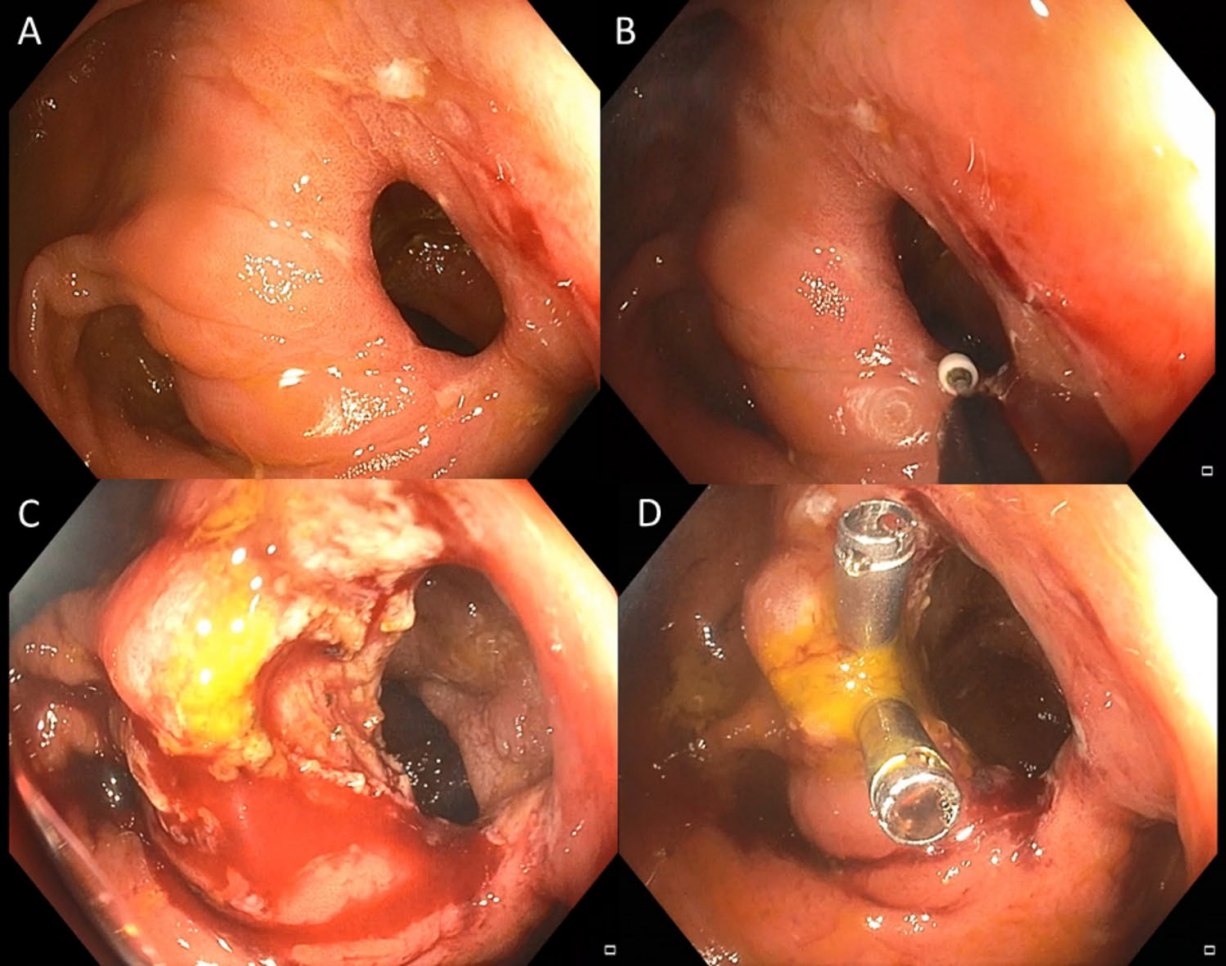
Endoscopic stricturotomy. **A** native stricture, **B** cutting with IT knife, **C** result after cutting, **D** clips which secure the deepest cutting area

For ESt procedures, an isolated-tip knife nano (ITknife nano, Olympus, Japan) was employed to create short incisions measuring 5–10 mm. The site of the anastomotic cut was chosen according to the anastomosis configuration to minimize the risk of perforation, typically at the afferent or blind (efferent) loop. Following stricturotomy, the incision site was secured with endoscopic clips (Resolution clip, Boston Scientific, USA) to ensure hemostasis (Fig. 4).

### Follow-up and measurement

**Fig. 5:**
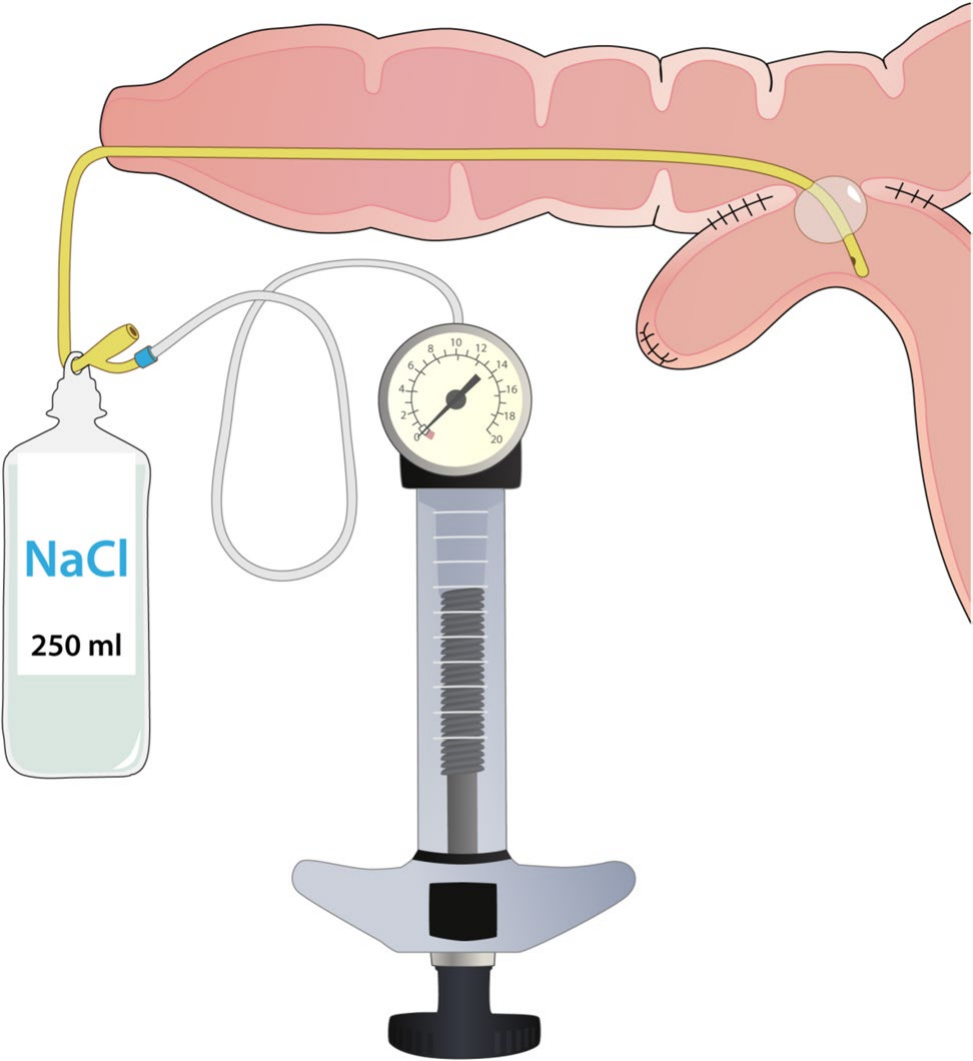
Scheme of anastomotic diameter measurement using Foley catheter and invariable pulling force. Deviation from the baseline diameter in each individual follow-up measurement

Throughout the study, veterinarians and caretakers closely monitored the health of animals in interventional and control groups, with body weight and any adverse events documented at every assessment. The anastomotic site was evaluated under short general anesthetic every 8 weeks up to 6 months. Stricture diameter was measured using a Foley urinary catheter connected to an endoscopic balloon inflation device. A standard 2-way 14Fr Foley catheter with 5–10 mL balloon (Teleflex, USA) was used for all diameter assessments. New catheter was used for each measurement to avoid potential discrepancies caused by deformation from repeated use. The catheter tip was grasped with endoscopic forceps through the working channel and guided through the stricture. The Foley balloon was filled with water until its diameter exceeded that of the stricture. Continuous mild tension was applied by suspending a 250-mL saline bottle from the external end of the catheter. The balloon diameter was then gradually reduced using the inflation device until it passed through the stricture (Fig. 5), and the resulting diameter was measured with a digital caliper.

### Statistics

Descriptive statistical analyses were performed, including the calculation of mean values, standard deviations, and/or ranges for continuous variables. Differences in diameters were evaluated using a non-parametric Mann–Whitney and Wilcoxon matched-pairs signed rank test. Statistical analyses were performed with GraphPad Prism (version 10.6.1), and a *p* value of less than 0.05 was considered statistically significant.

The minimum number of subjects was estimated based on expected outcomes according to Fleiss. In the original development of the animal model by the same team, the mean anastomotic diameter at 6 months was 11.7 ± 3.4 mm [9]. A dilation resulting in a 25% increase in diameter was considered clinically significant. With a one-sided significance level of 5% and a statistical power of 80%, the minimum group size was calculated as 14 subjects. Including additional animals would have conflicted with the 3R principles (Replacement, Reduction, and Refinement).

## Results

**Fig. 6:**
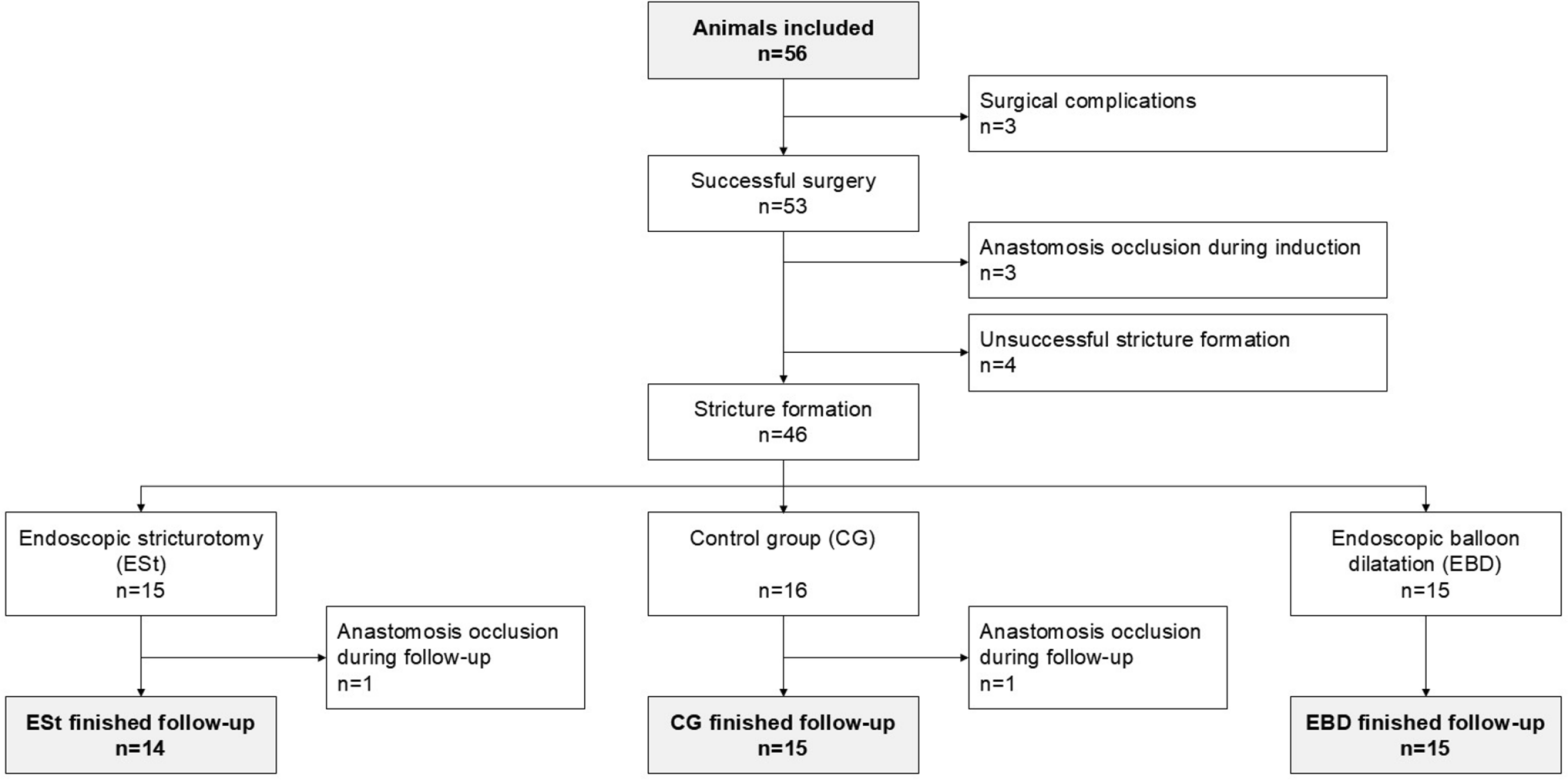
Flowchart of the study—enrollment, randomization, and follow-up

Fifty six minipigs (mean weight 48.5 ± 10.5 kg) underwent surgery. In three subjects, adverse postoperative events occurred including inadvertent inclusion of the urethra within the midline laparotomy closure that resulted in mechanical obstruction of urinary flow. These subjects were excluded from the further follow-up. Following a median of four injection sessions (range 3–7) administering 10.7 ± 2.9 mL of solution per session, an anastomotic stricture developed in 46 pigs. Stricture formation was unsuccessful in seven pigs: in four animals, the anastomosis remained patent even after up to seven injections, while in three other subjects, the anastomosis became completely occluded during induction. The 6-month follow-up was completed by 44 randomized subjects, when in 2 animals the anastomosis fully closed up during the follow-up period—one in the control group and one in the ESt group (Fig. 6).

### Animal welfare

Following surgery, three animals developed postoperative anuria due to inadvertent urethral entrapment during midline laparotomy closure, necessitating humane euthanasia in accordance with institutional animal-welfare guidelines. In the remaining animals, no notable alterations in physical condition or feeding behavior were observed after surgical or endoscopic interventions.

Additional complications included one transient rectal prolapse, which resolved spontaneously without treatment. During follow-up, two animal sustained an iatrogenic perforation after endoscopic balloon dilatation, and another animal developed a perforation after endoscopic stricturotomy; all defects were closed endoscopically (through-the-scope clips), and all three subjects continued in the study without further issues.

**Fig. 7:**
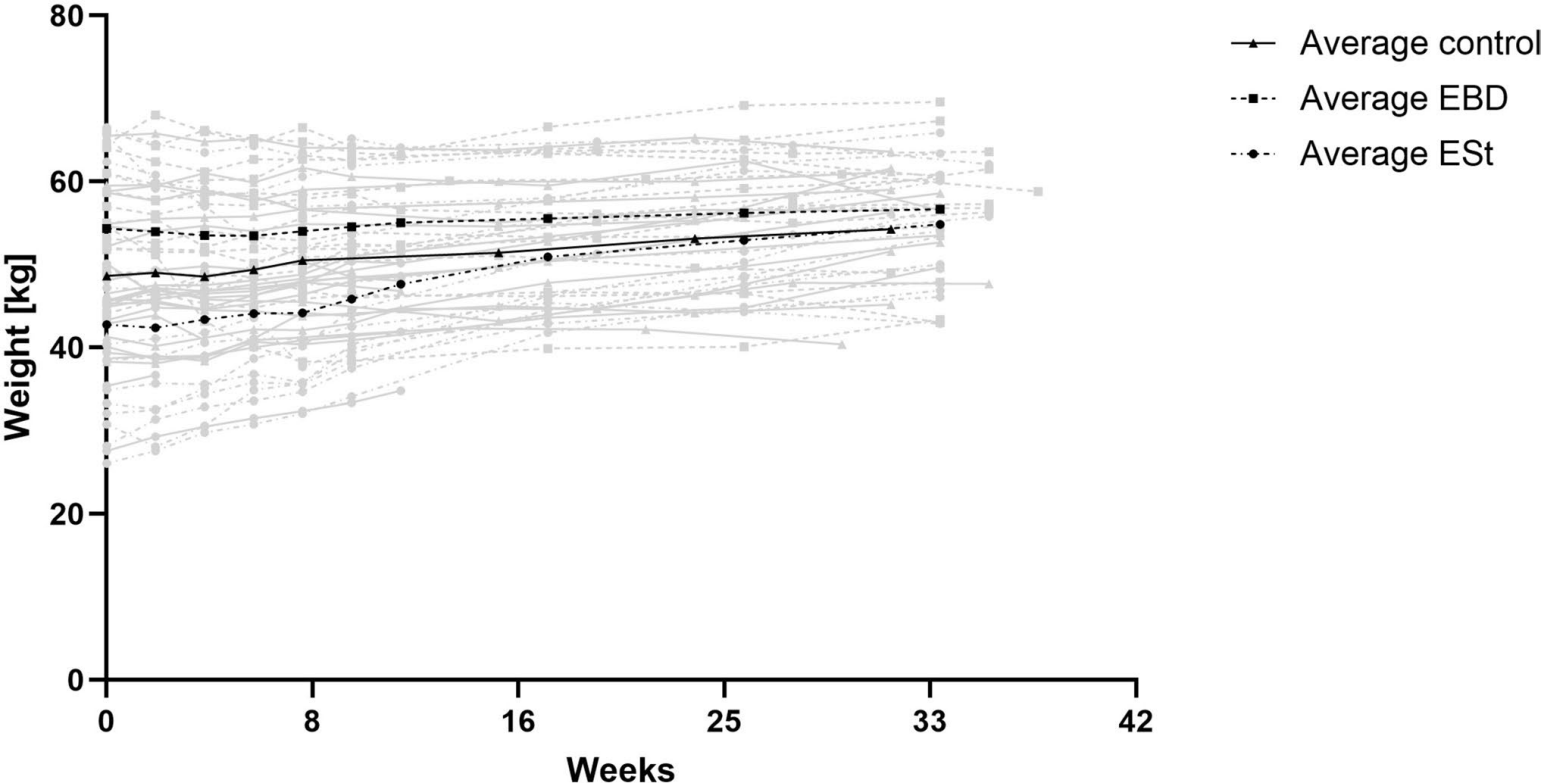
Weight increase in all cohorts over time of the study; *EBD* endoscopic balloon dilatation; *ESt* endoscopic stricturotomy

Baseline body weights differed across groups because animals were not stratified by weight at allocation. Control animals entered the study with a mean baseline weight of 48.65 ± 7.92 kg, similar to the overall cohort (48.08 ± 10.67 kg). The EBD group consisted mainly of fully mature animals with higher baseline weights (54.36 ± 7.19 kg), whereas the ESt group included younger, lighter animals (42.78 ± 12.53 kg). Across the study cohort, mean body-weight gain was 5.81 ± 8.35 kg, but growth patterns differed between groups: control animals gained 5.07 ± 5.39 kg, EBD animals showed minimal change (1.57 ± 6.36 kg), and ESt animals exhibited the greatest increase (11.14 ± 10.20 kg). These divergent trajectories are consistent with the differing maturational stages at baseline—adult animals in the EBD group exhibited little further growth, whereas younger ESt animals continued to physiologically mature. Despite baseline heterogeneity, final body weights converged across groups, with means of 54.27 ± 6.51 kg in controls, 56.67 ± 7.97 kg in EBD animals, and 54.84 ± 7.09 kg in ESt animals, indicating that these initial imbalances did not result in substantial differences in end-of-study body mass (Fig. 7).

### Endoscopic interventions

**Fig. 8:**
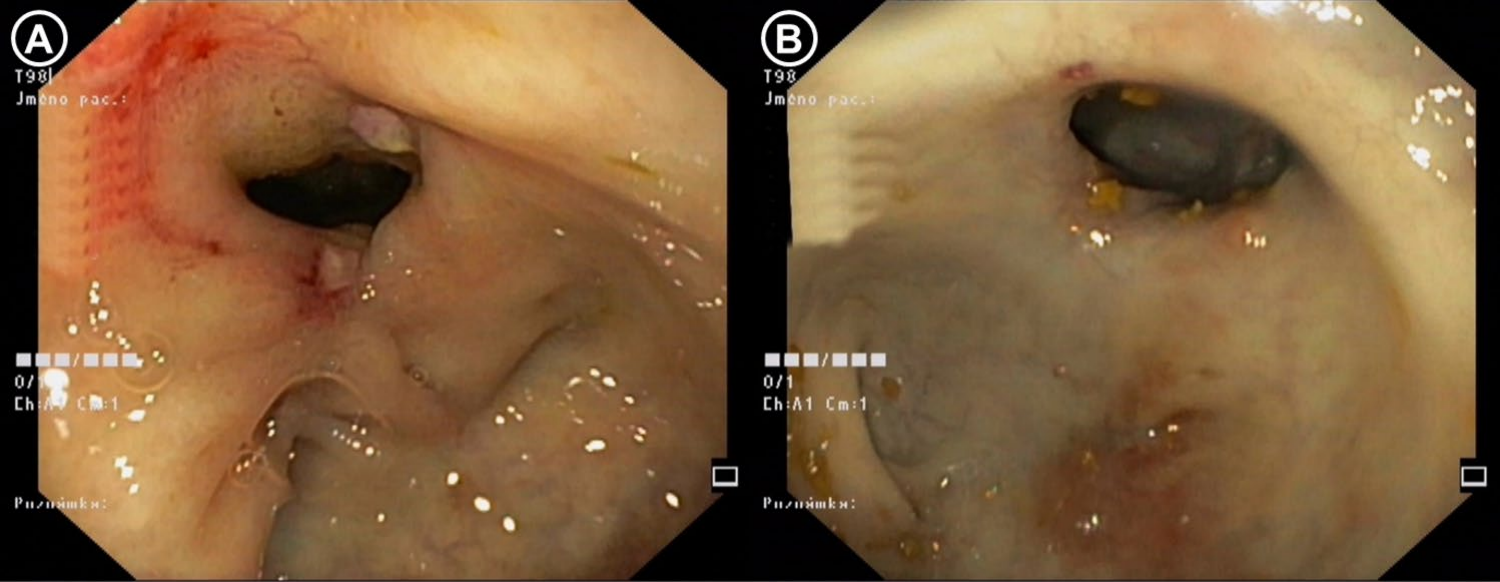
Endoscopic findings at baseline before intervention (**A**) and 6 months after EBD (**B**)

**Fig. 9:**
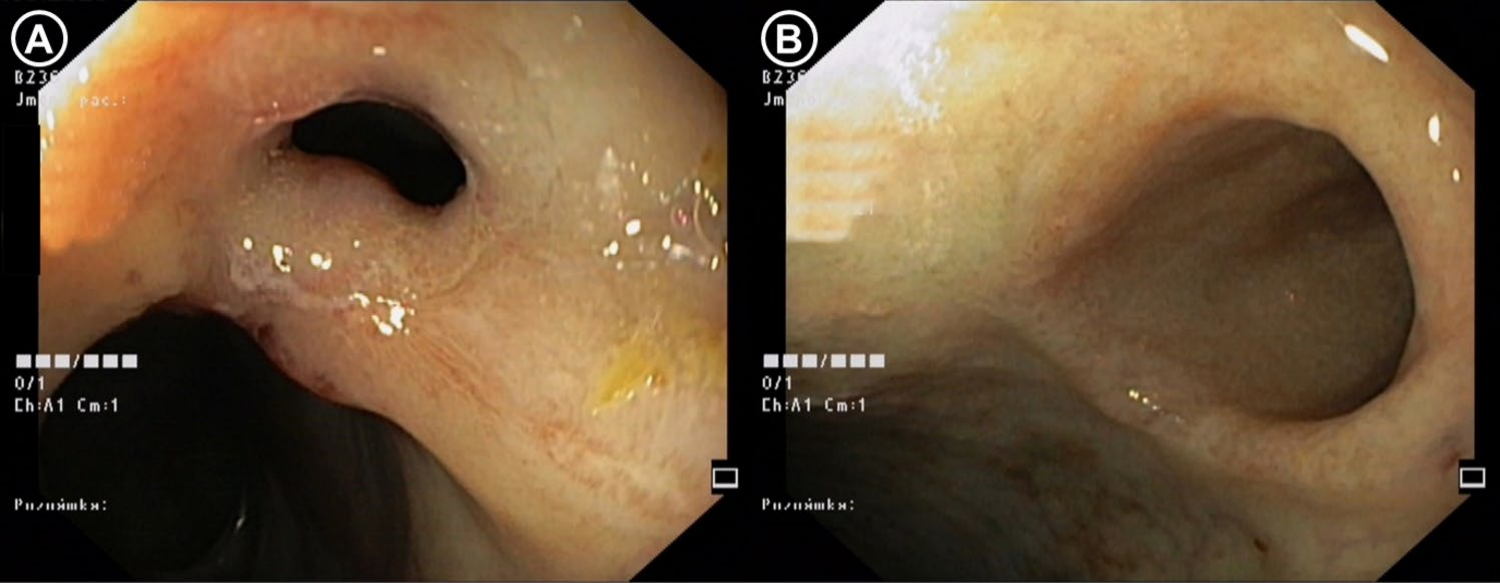
Endoscopic findings at baseline before intervention (**A**) and 6 months after ESt (**B**)

**Fig. 10:**
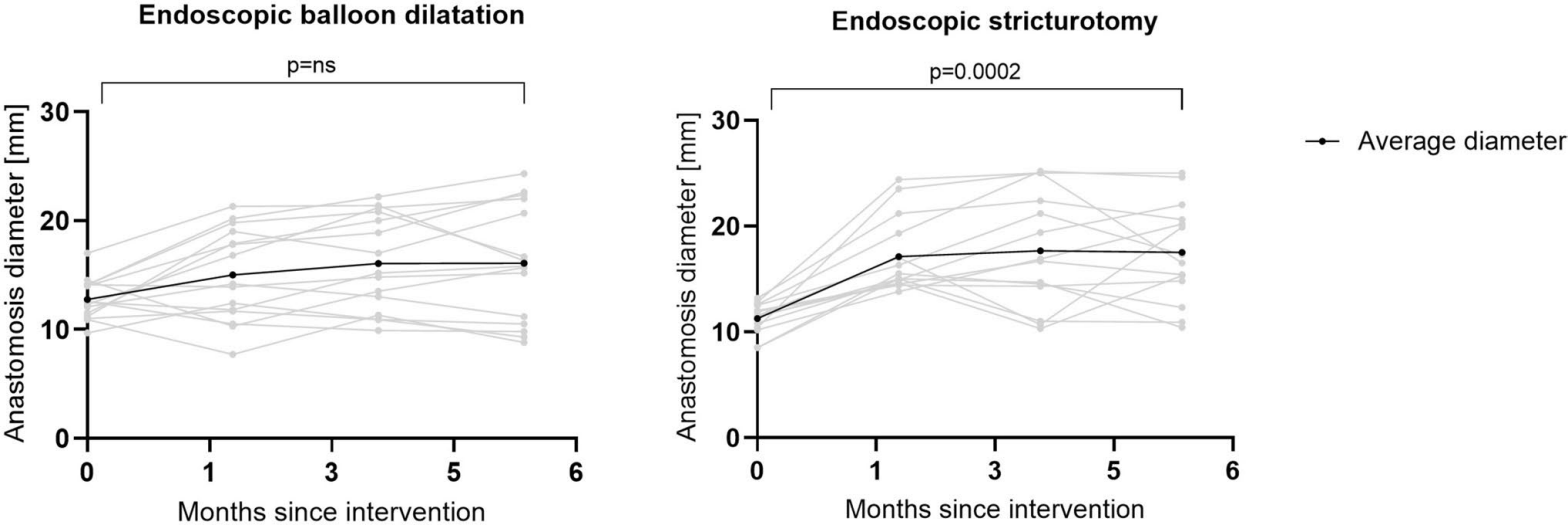
Change in diameter of the anastomosis during follow-up period after intervention

Baseline stricture diameter was comparable between the cohorts with 12.77 ± 1.86 mm in EBD group and 11.27 ± 1.46 mm in ESt group (*p* = ns). Including the subjects with fully completed follow-up, subjects in both interventional groups demonstrated an increase in anastomotic diameter compared with the post-induction baseline, which was maintained throughout the 6-month follow-up period, however, only reached statistical significance in the ESt group (Figs. 8, 9). The final stricture diameters were 16.09 ± 5.35 mm in EBD group (*p* = 0.0554) and 17.51 ± 4.71 in ESt group (*p* = 0.0002) (Fig. 10).

**Fig. 11:**
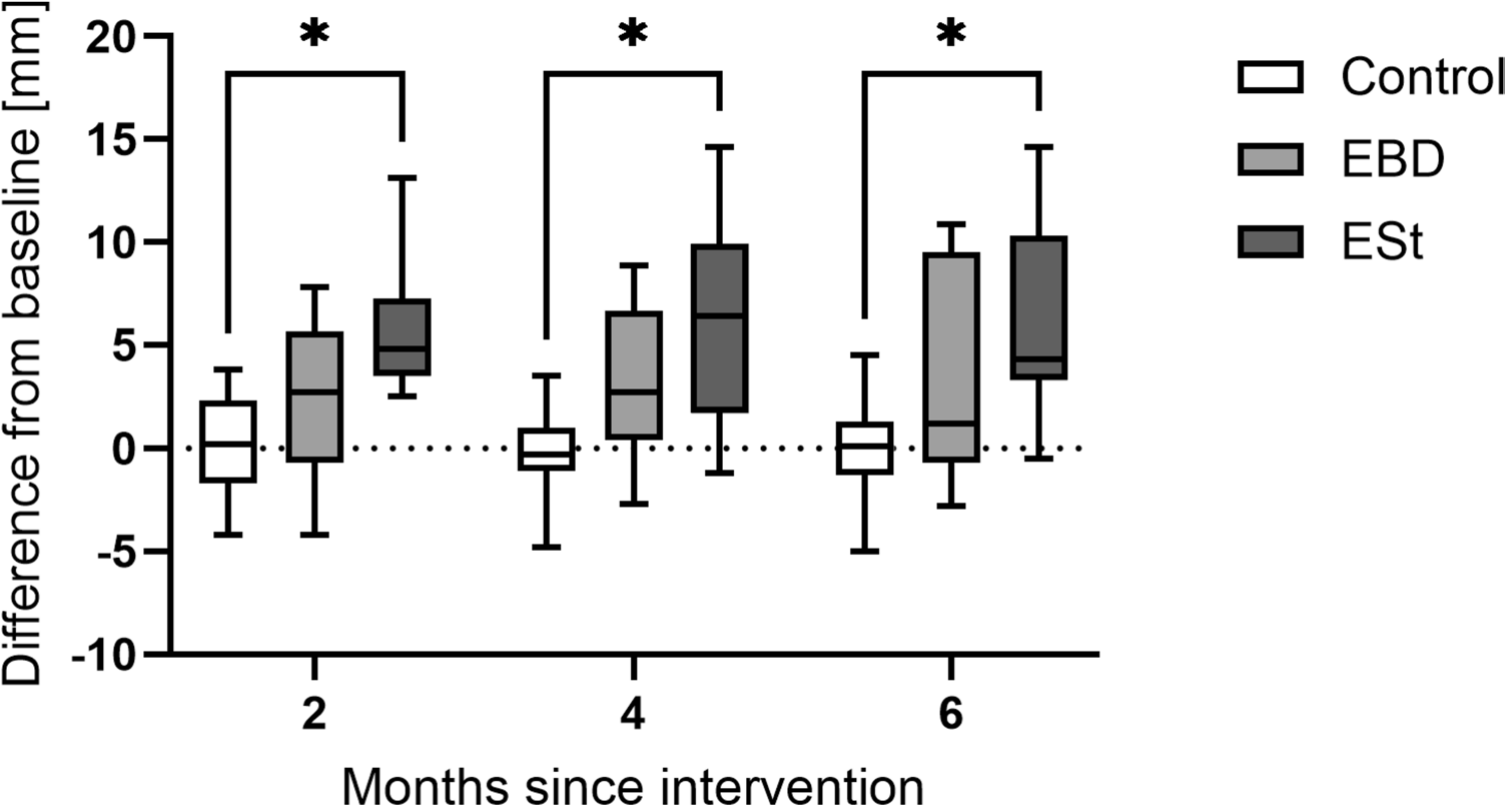
Difference in diameter of the anastomosis from baseline during follow-up breakpoints in all cohorts; *EBD* endoscopic balloon dilatation; *ESt* endoscopic stricturotomy

Stricture diameter in the control group was also similar to both interventional cohorts with mean of 11.81 ± 2.15 mm and remained stable during the whole follow-up period with concluding diameter averaging 11.67 ± 3.42 mm (*p* = ns). Differences relative to the control group were significant at 2, 4, and 6 months post-intervention in the ESt group, but not in the EBD group (Fig. 11).

The mean increase in anastomotic diameter from pre-intervention to 6 months post-intervention was significantly greater in the ESt group compared with the EBD group (6.24 ± 4.40 mm vs. 3.31 ± 5.01 mm; *p* = 0.042). In both interventional groups, the increase in diameter exceeded that observed in the control group.

## Discussion

Complications associated with CD such as strictures frequently necessitate surgical intervention. Postoperative disease recurrence is common following bowel resection with anastomosis. The incidence of secondary or anastomotic strictures varies widely, ranging from 3 to 30%, depending on the underlying disease, the surgical approach, and the anastomotic site [10]. Despite advancements in surgical techniques, stricture recurrence—including after stricturoplasty—remains high. Although surgery has long been the standard treatment for IBD-related strictures, endoscopic techniques have increasingly gained recognition as effective therapeutic alternatives. The primary objective of endoscopic management is to prevent, delay, or reduce the frequency of major surgical interventions [11, 12].

Although some clinical evidence supporting the superiority of ESt over EBD has been published, it is largely derived from case series and observational studies. Lan et al. compared a historical EBD cohort with a small group of patients treated using a newly established ESt technique, with limited follow-up. Furthermore, a substantial proportion of patients undergoing ESt had previously failed balloon dilation. The authors reported a high risk of bias and emphasized the need for randomized trials [7]. In this context, a comparison using a standardized animal model may reduce confounding factors and minimize bias inherent in clinical observational studies.

This study represents the first—and to our knowledge the only—prospective randomized experimental trial directly comparing the two most commonly used endoscopic techniques for managing anastomotic strictures. Our data support the feasibility and efficacy of endoscopic treatment strategies in management of anastomotic stricture associated with Crohn’s disease. All strictures were narrow and not passable before interventions. By evaluating these approaches within a controlled experimental setting, it provides a level of comparative evidence that has not previously been available. Importantly, the trial was conducted using a unique and fully standardized experimental model specifically designed to mimic anastomotic stricture formation associated with Crohn’s disease. Our model combines construction of a surgically narrowed anastomosis with serial intramural injections of TNBS and liquefied phenol was used [9]. Ziv et al. demonstrated that intramural phenol injection in porcine bowel induces a Crohn’s-like histopathological inflammatory response [13]. The addition of TNBS, which acts as a hapten and triggers a T-cell–mediated immune reaction, was intended to further replicate key inflammatory mechanisms involved in Crohn’s disease–related stricture formation [14]. This approach was adapted by using liquefied phenol with titrated TNBS in an aqueous solution injected at the ileocolonic anastomosis. Repeated low-dose injections were employed to induce progressive fibrosis while minimizing excessive tissue necrosis. Histopathologic analysis of resected specimens confirmed that the model reproduces key features of Crohn’s disease–associated fibrostenotic strictures, including severe submucosal fibrosis, chronic inflammation with lymphoplasmacytic and eosinophilic infiltrates, and the presence of histiocytic granulomas with multinucleated giant cells and epithelioid microgranulomas. We demonstrated that the anastomotic lumen remained stable for at least 6 months, which we considered essential to minimize confounding factors and enable meaningful randomized comparisons [9].

This model has not been used in prior studies and therefore offers a novel platform for assessing endoscopic therapies under controlled conditions [9, 15].

Evidence on more intensive endoscopic treatment modalities for Crohn’s disease–related strictures remains limited. In an uncontrolled study evaluating ESt, all five patients achieved technical success, although follow-up was short [16]. Similarly, an uncontrolled retrospective series reported technical success in all cases, but 60% of patients required repeat intervention at a median follow-up of 11 months [12].

As with EBD, the principal safety concerns associated with ESt relate to the risks of perforation and significant bleeding. In Crohn’s disease-related intestinal and anastomotic strictures, pooled clinical series of EBD report major complication rates of roughly 2–5% per procedure, with perforation rates of about 1–5% and clinically relevant bleeding in 3–5% of dilations [17–19]. Data for ESt are more limited but suggest a low perforation risk of approximately 1% and somewhat higher rates of procedure-related bleeding (around 6–10%) while maintaining excellent technical and clinical efficacy [20, 21]. Zhang et al. reported bleeding in 4.7% of ESt procedures for IBD-associated anastomotic strictures with no perforations [5], and the European experience by Lukáš et al. (92 procedures in 67 patients) similarly demonstrated only delayed bleeding events—each managed conservatively—and likewise no perforations [6].

In our porcine model, we observed two transmural perforations after EBD and one perforation after ESt, all immediately recognized and successfully closed endoscopically with clips. No procedure-related mortality or clinically relevant bleeding occurred, and all affected animals continued in the protocol. Although these events appear more frequent than in clinical practice, this likely reflects the limited sample size and the deliberately narrow, fibrotic anastomotic strictures created experimentally rather than a fundamentally different risk profile.

This study has several limitations. Although the experimental model was designed to reproduce key features of Crohn’s disease–related anastomotic strictures, it does not fully replicate the complexity of human disease. Nonetheless, the model successfully generated both inflammatory changes through chemical induction and subsequent fibrotic remodeling, which were confirmed histologically [9]. Importantly, the controlled experimental setting avoided the substantial variability encountered in clinical practice, including differences in anastomotic configurations, patient-specific disease behavior, and concomitant medications. This level of standardization enhances the precision of comparing the two endoscopic techniques, a comparison that would be extremely difficult—if not impossible—to perform reliably in a heterogeneous patient population.

In conclusion, both EBD and ESt are safe and effective therapeutic approaches for managing IBD-associated anastomotic strictures. However, stricturotomy appears to offer greater efficacy and more durable long-term outcomes. Findings from this experimental study support its potential to delay—and in some cases avoid—the need for stricture-related surgical intervention.
